# Brain development is impaired in *c-fos* −/− mice

**DOI:** 10.18632/oncotarget.4527

**Published:** 2015-06-19

**Authors:** Fabiola N. Velazquez, César G. Prucca, Olivier Etienne, Diego S. D'Astolfo, David C. Silvestre, François D. Boussin, Beatriz L. Caputto

**Affiliations:** ^1^ CIQUIBIC (CONICET), Departamento de Química Biológica, Facultad de Ciencias Químicas, Universidad Nacional de Córdoba, Córdoba, Argentina; ^2^ Laboratoire de Radiopathologie, CEA, Institut de Radiobiologie Cellulaire et Moléculaire, Fontenay-aux-Roses, France; ^3^ INSERM UMR967, Fontenay-aux-Roses, France; ^4^ Université Paris VII, UMR967, Fontenay-aux-Roses, France; ^5^ Université Paris XI, UMR967, Fontenay-aux-Roses, France

**Keywords:** apoptosis, differentiation, neocortex, neural stem progenitor cells (NSPCs), neurogenesis

## Abstract

c-Fos is a proto-oncogene involved in diverse cellular functions. Its deregulation has been associated to abnormal development and oncogenic progression. *c-fos−/−* mice are viable but present a reduction in their body weight and brain size. We examined the importance of c-Fos during neocortex development at 13.5, 14.5 and 16.5 days of gestation. At E14.5, neocortex thickness, apoptosis, mitosis and expression of markers along the different stages of Neural Stem Progenitor Cells (NSPCs) differentiation in *c-fos−/−* and wild-type mice were analyzed. A ∼15% reduction in the neocortex thickness of *c-fos−/−* embryos was observed which correlates with a decrease in the number of differentiated cells and an increase in apoptosis at the ventricular zone. No difference in mitosis rate was observed, although the mitotic angle was predominantly vertical in *c-fos−/−* embryos, suggesting a reduced trend of NSPCs to differentiate. At E13.5, changes in differentiation markers start to be apparent and are still clearly observed at E16.5. A tendency of more AP-1/DNA complexes present in nuclear extracts of cerebral cortex from *c-fos−/−* embryos with no differences in the lipid synthesis activity was found. These results suggest that c-Fos is involved in the normal development of NSPCs by means of its AP-1 activity.

## INTRODUCTION

Accumulated evidence indicates that proto-oncogenes that are implicated in controlling cell proliferation may have additional roles in differentiation and development [1, 2]. Such is the case of c-Fos, a well-known immediate early response proto-oncogene described more than 30 years ago as a transcription factor [3]. c-Fos has been implicated in differentiation [4, 5], proliferation [6], and malignant cell transformation [7-9] in response to diverse mitogenic stimuli. Consistent with the importance of c-Fos for development is the 40-60% smaller size of *c-fos −/−* adult mice, a phenotype that has been also evidenced at embryonic day 15.5 [10]. Furthermore, only ∼40% of *c-fos −/−* embryos survive until birth and surviving mice live to an average age of 6-7 months, show growth retardation, severe osteopetrosis, delayed or absent gametogenesis, altered hematopoiesis and abnormal behavior [10, 11], although the development of the Central Nervous System (CNS) of these *c-fos −/−* mice has not been studied up to date. However, it is worth highlighting the observation that *c-fos −/−* mice are viable thus evidencing that c-Fos, although important, is not essential for mouse development.

c-Fos contains two domains: a basic domain (BD) and a leucine zipper domain (LZ). The BD is responsible for DNA-binding whereas the LZ is a leucine-repetition region involved in heterodimerization of c-Fos with other leucine zipper-containing proteins, mainly c-Jun, thus constituting the AP-1 family of transcription factors [12]. Although c-Fos does not form homodimers [12], as an AP-1 heterodimer c-Fos is considered a master switch that transduces short-term stimuli into long-term responses [3, 12]. Constitutive expression of c-Fos has also been reported in adult bone marrow and growing bone [13] whereas in adult brain, c-Fos expression has been used as a marker of neuronal activity [14].

An additional activity has been described for c-Fos which is independent of its AP-1 activity: at the endoplasmic reticulum, c-Fos associates with and activates key enzymes of the pathway of synthesis of lipids thus promoting an overall activation of lipid synthesis and consequently, of membrane biogenesis [5, 15]. This phenomenon occurs in processes that demand high rates of membrane biogenesis such as differentiation [5, 16], and normal and exacerbated proliferation [17-19].

Early reports showed the need of c-Fos for normal differentiation of cultured PC12 rat cells into sympathetic neuron-like cells [4, 5] and for the regulation of neuronal excitability and survival [20], among other neuronal functions [14]. Based on these observations and taking into account that several pathologies are related with an impairment in the normal development of the neocortex [21] we studied the importance of c-Fos during CNS development in mice, focusing on the consequence of its lack of expression in the content and fate of NSPCs. A significant decrease of 20% in the differentiation rate of NSPCs into mature neuronal cells was found in *c-fos −/−* embryos as compared to *c-fos +/+* ones. This decrease correlates with an increase in the content of AP-1 transcription factors in the developing CNS with no evident changes in the rate of lipid synthesis. These observations suggest that c-Fos is involved in the initial differentiation of NSPCs during neocortex development as a classical AP-1 transcription factor rather than as an activator of lipid synthesis.

## RESULTS

### Neocortex size is reduced in *c-fos −/−* embryos

**Figure 1:**
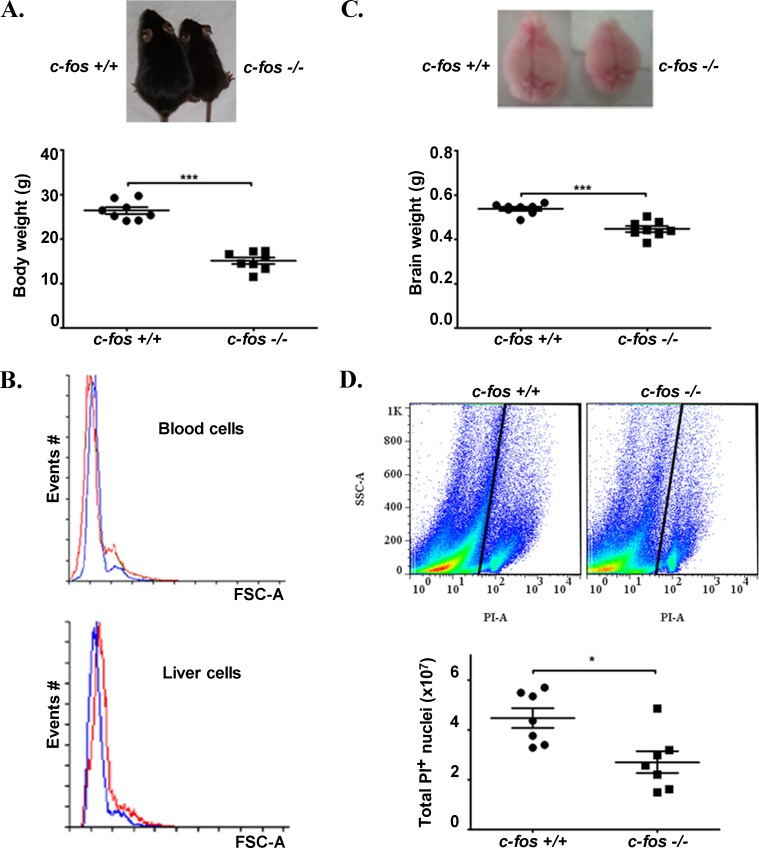
Adult *c-fos* −/− mice are smaller, their brains weigh less and their cerebral cortex contains fewer cells than that of *c-fos* +/+ ones **A.** Two two-month-old littermate mice, *c-fos* +/+ and *c-fos* −/− as indicated are shown (upper panel). The body weight of the two-month-old adult *c-fos* −/− mice is significantly smaller than that of *c-fos* +/+ mice (lower panel). Results are shown as the mean body weight (grams) ± SEM (*n* = 8) **B.** Blood (upper panel) and liver cells (lower panel) from *c-fos* +/+ (blue line) and *c-fos* −/− (red line) mice were analyzed by flow cytometry and their size (FSC-A) compared. The histograms show the number of events vs FSC-A. **C.** The brain weight of two-month-old adult *c-fos* −/− mice was significantly smaller than that of *c-fos* +/+ mice. Results are shown as the mean brain weight (grams) ± SEM (*n* = 8). **D.** Nuclei isolated from adult cerebral cortex were stained with PI and positive cells quantified by flow cytometry. Scatterplots (top panel) show the nuclei events on a SSC-A (Side Scatter Area) vs. PI-A. A polygon gate defined as stated under Materials and Methods was used to select the nuclei based on PI staining. This analysis profile allows the quantification of singlet nuclei in a known volume of sample and excludes the debris present in the left edge of the plot. Quantification of the number of cell nuclei present in the cerebral cortex of adult *c-fos* +/+ and *c-fos* −/− mice was performed (lower panel). Results are shown as the mean number of PI positive nuclei ± SEM of 7 independent experiments. *p < 0.05, **p < 0.01; ***p < 0.001 in *c-fos* −/− animals with respect to the c-fos +/+ condition using unpaired t test with Welch's correction.

Adult *c-fos −/−* mice present a ∼ 40-60% reduction in their body weight as compared to *c-fos +/+* mice [10] (Figure 1*A*). Considering the two previously described activities of c-Fos that are, as a transcription factor and as an overall activator of lipid synthesis/membrane biogenesis, a simple explanation for this difference could be that *c-fos* −/− mice are lacking the c-Fos-dependent activity of lipid synthesis to enable high rates of membrane biosynthesis, which could determine either a reduction in the size that ­*c-fos −/−* cells reach, or a decrease in the number of cells present in the adult animal. An alternative explanation is that the AP-1 transcription factor activity is promoting changes in the expression of genes that determine the reduction in cell size or number. In either case, results indicate that this reduction in the size of *c-fos −/−* animals is due to a decrease in the number of cells contained in their organs and tissues rather than to a content of smaller cells because the mean size of adult blood and liver cells do not differ between adult *c-fos −/−* and *c-fos +/+* mice (Figure 1*B*).

Adult *c-fos −/−* animals also show a marked reduction in their brain weight (Figure 1*C*). In line with these differences is the finding of a clear reduction in the thickness of the cerebral cortex in *c-fos −/−* adult mice as compared to their *c-fos +/+* littermates (Supplementary Figure 1). To confirm that, again, cell number rather than size is promoting these differences, cells present in the adult cerebral cortex were labeled with PI and PI positive nuclei quantified by flow cytometry. The cerebral cortex of 2-month-old *c-fos −/−* mice contained a significantly lower number of cells (∼40%) as compared to their *c-fos +/+* littermates (Figure 1*D*).

**Figure 2:**
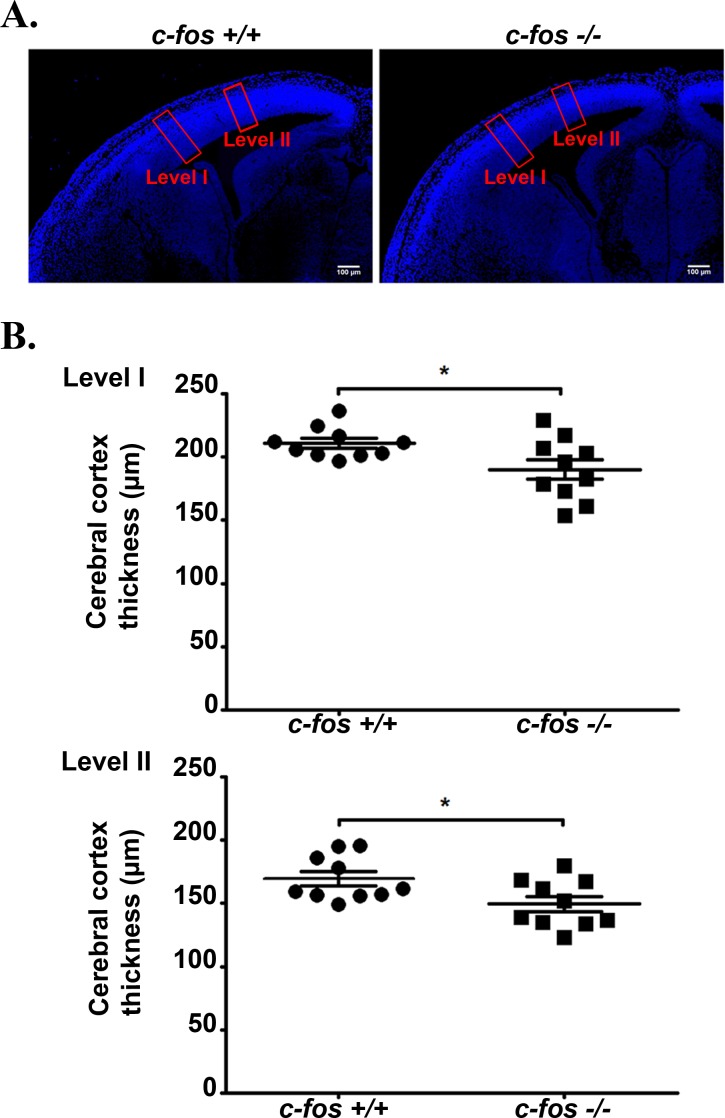
The neocortex of *c-fos* −/− embryos is thinner than that of their *c-fos* +/+ counterpart **A.** Fixed cerebral cortex of E14.5 *c-fos* +/+ and *c-fos −/−* embryos were stained with DAPI and examined under a confocal microscope. Boxes in red represent the areas used for the quantifications shown in B. **B.** The thickness of the neocortex was determined by measurements performed at two different right-left levels (I and II) for each genotype slice as marked in A. Four determinations were done in each sample; 10 samples of each genotype were examined. Results shown are the mean thickness of neocortex (μm) ± SEM. *p < 0.05 in *c-fos* −/− embryos with respect to the *c-fos* +/+ littermates using unpaired t test with Welch's correction.

Based on the above observations and taking into consideration that the NSPCs of the developing neocortex give rise to the majority of the cells of the adult cerebral cortex, the behavior of this population of cells was evaluated in *c-fos +/+* and *c-fos −/−* embryos. For this, we first analyzed the total thickness of the neocortex at 14.5 days of gestation (E14.5), a developmental stage in which high rates of neurogenesis are observed [22]. Coronal sections of the embryo brain were stained with DAPI, visualized under a fluorescence microscope and neocortex thickness measured at two different levels (Figure 2A). Neocortex thickness at both levels I and II of *c-fos −/−* embryo is significantly smaller than that from *c-fos +/+* littermates analyzed in parallel (Figure 2B). These differences could be due either to a decrease in mitosis in the *c-fos −/−* embryos, to an increase in apoptosis in these embryos, to impairment in the differentiation/migration of NSPCs or to the concomitant occurrence of more than one of these events.

### Mitotic events are similar in *c-fos* −/− and *c-fos* +/+ embryo

**Figure 3:**
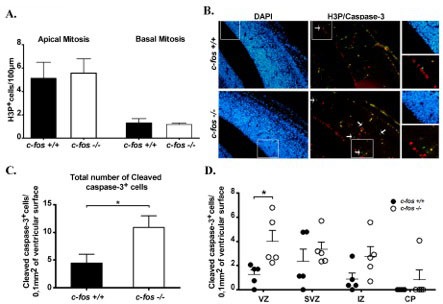
Mitosis and apoptosis in the neocortex **A.** Paraffin coronal sections of the neocortex were stained with the mitotic marker Phospho Histone H3 (H3P). H3P positive nuclei/100μm of length of the neocortex ventricular surface were quantified separately according to the mitosis they undergo as: apical mitosis (left panel) and basal mitosis (right panel). Results are the mean number of H3P positive nuclei/100μm ± SEM from 6 embryos of each genotype. No significant differences were observed between *c-fos* +/+ and *c-fos −/−* conditions. **B.** Photomicrographs for DAPI (blue), H3P (red) and cleaved caspase-3 (green) of brain coronal sections obtained from E14.5 *c-fos* +/+ and *c-fos* −/− embryos. Arrows indicate cells undergoing apoptosis (cleaved caspase-3 positive cells). Images were obtained with a fluorescence microscope (Olympus BX51) using a 40X objective. Magnification of boxed area is shown in the third column. **C.** Quantification of the total number of cleaved caspase-3 positive cells/0.1 mm^2^ of developing cerebral cortex surface for *c-fos* +/+ (black bar) and *c-fos* −/− (white bar) embryo. **D.** Quantification of the cleaved caspase-3 positive cells /0.1 mm^2^ of the cerebral cortex areas detailed below in *c-fos* +/+ (black circles) and *c-fos* −/− (white circles) mice: ventricular zone (VZ), sub-ventricular zone (SVZ), intermediate zone (IZ) and cortical plate (CP). Results are the mean number of the cleaved caspase-3 positive cells /0.1 mm^2^ cerebral cortex ± SEM from 4 embryos of each genotype. *p < 0.05 in *c-fos* −/− embryos with respect to the *c-fos* +/+ condition using unpaired t test with Welch's correction (mitosis analysis) and two way ANOVA with Bonferroni post-test (apoptosis analysis).

To determine if mitosis impairment in the *c-fos −/−* animals contributes to establish the differences observed in the weight their brain reaches in adults, the number of mitotic cells present in *c-fos −/−* and *c-fos* +/+ developing cerebral cortex was determined by immunostaining of paraffin coronal sections of the neocortex with the mitotic marker Phospho Histone H3 (H3P) [23, 24]. When quantifying the positive cells, these were distinguished between cells undergoing apical mitosis (at the ventricular surface of the neuroepithelium) or basal mitosis (non-surface mitosis). No significant differences were found in the number of mitotic events, either apical or basal, between *c-fos −/−* and *c-fos* +/+ embryos (Figure 3A), suggesting that the reduced number of cells found in the cerebral cortex of adult *c-fos −/−* mice was not due to differences in mitosis between both groups of animals.

### Apoptotic events are higher in *c-fos* −/− embryos

Apoptosis levels were examined by immunostaining of coronal slices of the neocortex with anti-cleaved capase-3 and quantification of positive cells that, in addition, evidenced a pyknotic nucleus. The total number of cleaved capase-3 positive cells increased in *ko* mice (Figure 3C). According to their location in the neocortex, cells were assigned to the ventricular zone (VZ), the sub-ventricular zone (SVZ), the intermediate zone (IZ) or the cortical plate (CP). More apoptotic cells were found in the VZ of c-*fos* −/− mice as compared to their *c-fos +/+* littermates (Figure 3B and quantification in Figure 3D) indicating that increased cell death at the VZ contributes to the decreased content of cells in the adult brain of c-*fos* −/− mice.

### Distribution of radial glial cells in the developing brain of *c-fos* −/− mice

**Figure 4:**
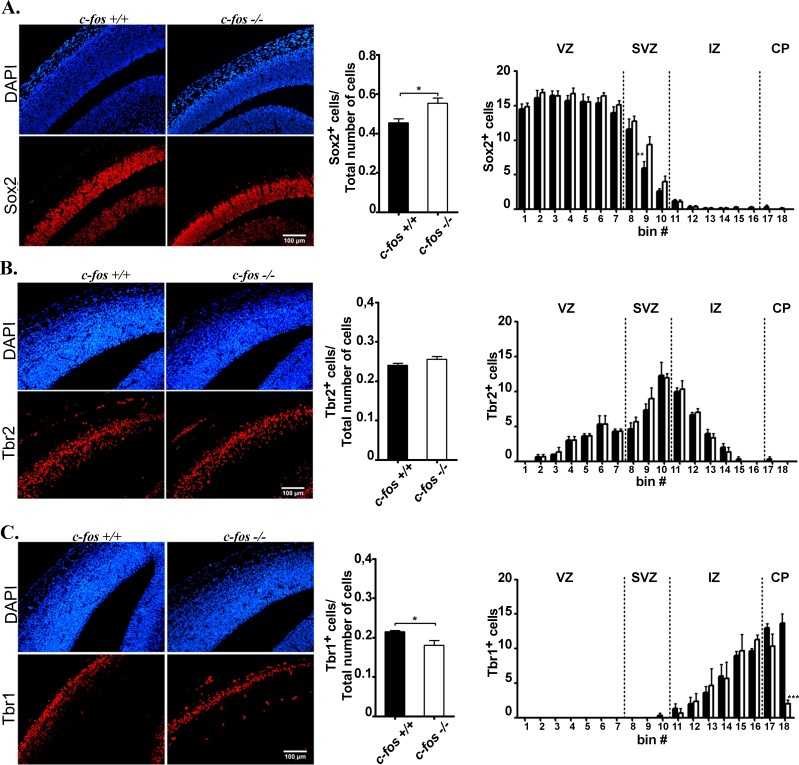
Cerebral cortex from E14.5 *c-fos* +/+ and *c-fos* −/− embryos differ in their cell populations Cortical slices staining for DAPI (blue) were analyzed in standard sectors of the dorso-medial cerebral wall. Segments of 100μm in the medial-lateral dimension were divided into 18 bins of 10 μm in height in its radial dimension. The sector was aligned such that the first bin was at the ventricle (V) surface with its long axis parallel to the ventricle border. Representative photomicrographs of the fluorescent staining shown in red (bottom row) for Sox2 **A.** Tbr2 **B.** and Tbr1 **C.** in brain coronal sections from the dorso-medial cerebral wall of *c-fos* +/+ and *c-fos* −/− embryos (left panel) are shown. Samples were stained for DAPI (blue, top row of A, B and C) and images obtained with a fluorescence microscope (Olympus BX51) using a 40X objective. The histograms show the quantification of the number of positive nuclei/total number of cells corresponding to Sox2 **A.**, Tbr2 **B.** and Tbr1 **C.** (middle panel) and the quantification of the number of positive nuclei/bin for Sox2 **A.**, Tbr2 **B.** and Tbr1 **C.** (left panel) staining respectively in brain coronal sections of E14.5 *c-fos* +/+ (black bars) and *c-fos −/−* (white bars) embryos, as indicated. Neocortex regions as shown in Figure 3D (VZ, SVZ, IZ and CP) are indicated. Results are the mean number of positive cells for each marker ± SEM from 4 sections of the 4 embryos examined per genotype. **p < 0.01; ***p < 0.001 in *c-fos −/−* samples with respect to the *c-fos* +/+ condition as determined by two-way ANOVA with Bonferroni post-test.

NSPCs, the stem/progenitor cells that give rise to neuronal and glial cells of the nervous system, are defined by their proliferation capacity and by their self-renewal and differentiation potential. Within the neocortex, two main types of progenitor cells can be distinguished during embryonic development: the radial glia cells that are less differentiated and, as development progresses, the intermediate progenitor cells (IPCs) [25]. The immunostaining of these two cell populations was analyzed in brain coronal sections prepared from E14.5 *c-fos +/+* and *c-fos −/−* embryos. Sox2 antibody was used as a radial glia cell marker and an early IPC marker [26, 27], whereas Tbr2 antibody was used as an IPC marker [25, 28]. To quantify immunostaining, a grid that optically divides the neocortex into 18 bins of 10μm x 100μm each, was used (Supplementary Figure 2). The Sox2 positive cells were analyzed with respect to the total number of cells as determined by DAPI staining, in the area of 180 μm x100μm examined; it was found a significant increase in these cells in the *c-fos −/−* as compared to the *c-fos +/+* embryos (Figure 4A, middle panel). In addition, when Sox2 expression was examined in the different neocortex zones, interestingly, no significant differences were observed in the VZ whereas more Sox2-positive cells are present in the SVZ (bins 8 to 10) of *c-fos −/−* embryos (Figure 4A, right panel), suggesting that *c-fos* deficiency may impact neurogenesis. The lack of difference in Tbr2 expression between both groups of embryos (Figure 4B) indicates that the importance of *c-fos* deficiency may vary in function of the differentiation stage of neural progenitors.

### The amount of post-mitotic neurons is reduced in *c-fos* −/− embryos

The transition from IPC to post-mitotic neurons is marked by the down-regulation of Tbr2 and up-regulation of Tbr1 expression [25, 29]. To examine the population of post-mitotic neurons in the developing cerebral cortex of *c-fos −/−* and *c-fos +/+* embryo, we determined the pattern of expression of Tbr1 in E14.5 brain coronal slices. The positive zone for Tbr1 is thinner in *c-fos −/−* than in *c-fos* +/+ embryos. Moreover, the corresponding quantification evidenced a significant decrease in Tbr1 labeling with respect to the total number of cells quantified using DAPI (Figure 4C, middle panel). This decrease was marked by the absence of positive cells in bin 18 of the *c-fos −/−* condition (Figure 4C), suggesting a reduction in the amount of post-mitotic neurons present in the *c-fos −/−* embryos.

**Figure 5:**
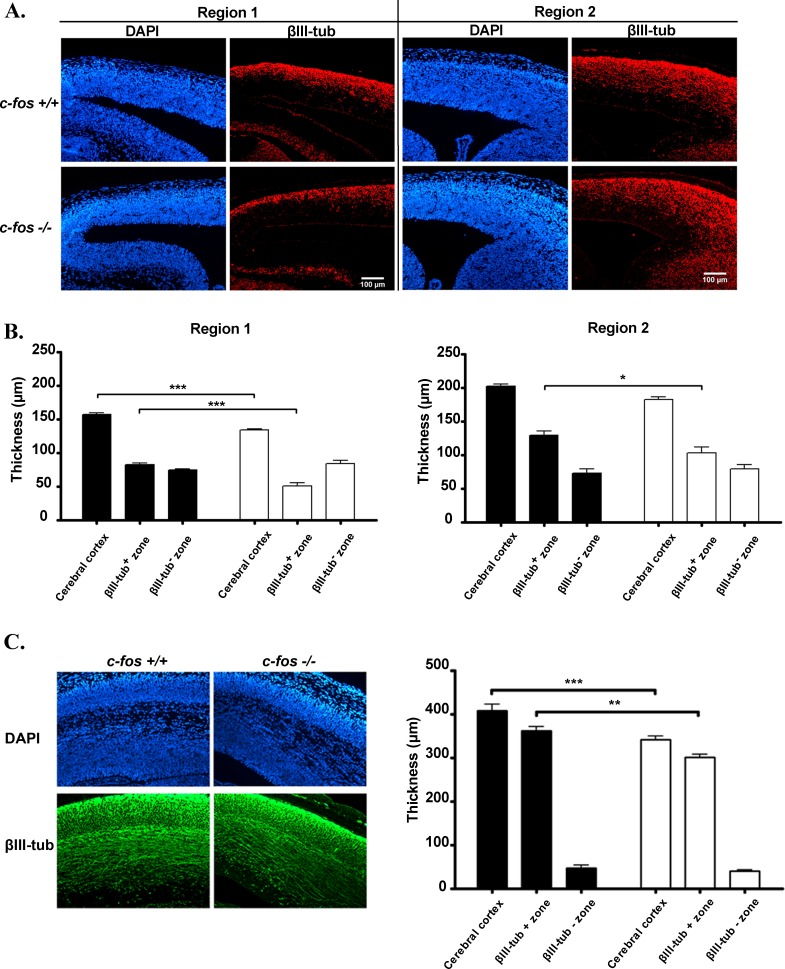
Neuronal differentiation is decreased in *c-fos* −/− embryo cerebral cortex **A.** Representative fluorescent photomicrographs of brain coronal sections obtained from two regions of the dorso-medial cerebral wall from E14.5 *c-fos* +/+ and *c-fos* −/− embryos stained for DAPI (blue) and βIII-tubulin (red). Images were obtained with a fluorescence microscope (Olympus BX51) using a 40X objective. **B.** Quantification of the thickness of the βIII-tubulin positive zone, βIII-tubulin negative zone and of the cerebral cortex of the regions shown in A, for *c-fos* +/+ (black bars) and *c-fos* −/− (white bars) embryos. **C.** Fluorescent photomicrographs for DAPI (blue) and βIII-tubulin (red) of brain coronal sections prepared from E16.5 *c-fos* +/+ and *c-fos* −/− embryos (left panel). Images were obtained with a fluorescence microscope (Olympus BX51) with a 40X objective. Quantification of the thickness of βIII-tubulin positive zone, βIII-tubulin negative zone and of the cerebral cortex of the figures shown (right panel), for *c-fos* +/+ (black bars) and *c-fos* −/− (white bars) embryos. Results are the mean thickness (μm) of the indicated zone ± SEM from 4 sections of 4 embryos of each genotype. *p < 0.05; **p < 0.01; ***p < 0.001 in *c-fos* −/− embryos with respect to the *c-fos* +/+ condition as determined by two-way ANOVA with Bonferroni post-test.

To confirm the reduction in the population of post-mitotic neurons in *c-fos −/−* embryos, another neuronal differentiation marker, βIII-tubulin (an early pan-neuronal marker) [30] was evaluated. A reduction in the thickness of the positive zone for this marker correlating with a thinner cerebral cortex was found in both regions examined of the dorso-medial cerebral wall of E14.5 *c-fos −/−* embryonic brain as compared with *c-fos +/+* controls (Figure 5A and 5B). By contrast, no significant differences were observed in the thickness of the βIII-tubulin-negative zones (Figure 5B).

In order to determine if the differences observed in βIII-tubulin staining of the cortical plate are shared by other brain regions and if these are long-lasting changes, βIII-tubulin staining was also examined both at later stages of development (E16.5) and in the whole brain. The difference in βIII-tubulin staining between *c-fos* +/+ and *c-fos −/−* embryos was evident at E16.5 (Figure 5C). Furthermore, the reduction of βIII-tubulin staining found in CP also concerned the whole brain of *c-fos −/−* embryos and not only the dorsal telencephalon as shown in Supplementary Figure 3 and Supplementary Figure 4.

### NeuN and MAP2 expression are reduced in *c-fos* −/− embryos

**Figure 6:**
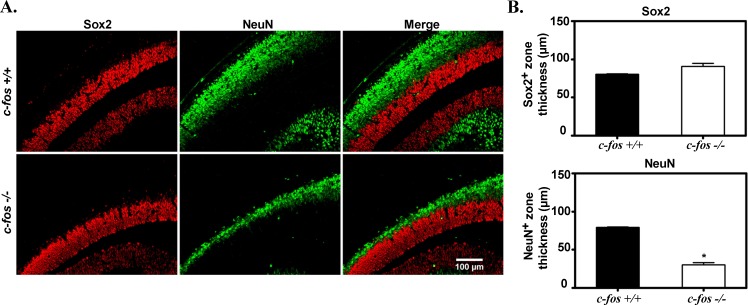
The NeuN positive zone of the cerebral cortex from *c-fos* −/− embryos is thinner than that of *c-fos* +/+ ones **A.** Representative photomicrographs of a region of the dorsomedial cerebral wall from each genotype stained for Sox2 (red) and NeuN (green). Images were obtained with a fluorescence microscope (Olympus BX51) using a 40X objective. **B.** Quantification of the thickness of the Sox2 and the NeuN positive zones of the regions shown in A, for *c-fos* +/+ (black bars) and *c-fos* −/− (white bars) embryos. The mean thickness (μm) ± SEM was calculated from 4 sections of 4 embryos of each genotype. *p < 0.05 in *c-fos* −/− embryos with respect to the *c-fos* +/+ ones as determined by unpaired *t*-test with Welch's correction.

The expression of NeuN, a nuclear protein widely expressed in mature post-mitotic neurons [31] was also analyzed together with the neuronal progenitor marker Sox2 on paraffin coronal sections of E14.5 *c-fos* +/+ and *c-fos −/−* embryonic neocortex. The Sox2 positive zone did not differ between both groups of embryo (Figure 6A, first panel and quantification in Figure 6B) whereas the thickness of the NeuN positive zone was significantly reduced in the *c-fos −/−* condition (Figure 6A second panel and quantification in Figure 6B).

**Figure 7:**
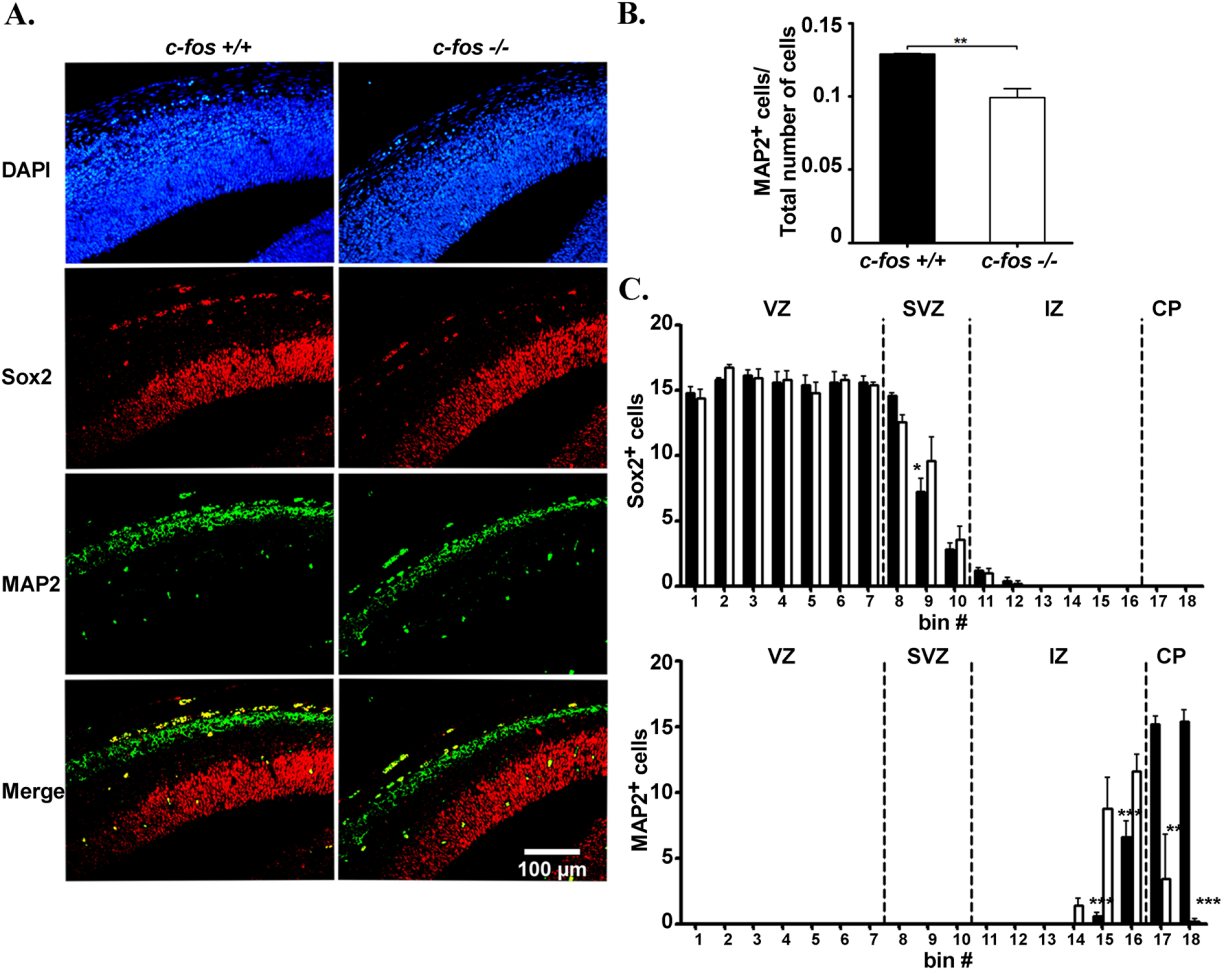
Analysis of Sox2 and MAP2 in the cerebral cortex of *c-fos* −/− embryo **A.** Immunostaining for DAPI (blue) (top row), Sox2 (red) (second row) and MAP2 (green) (third row) in the cerebral cortex from E14.5 *c-fos* +/+ and *c-fos* −/− embryos. The Sox2-MAP2 merged images are show (bottom row). **B.** Quantification of the number MAP2 positive cells/total number of cells in a region of 100μm x180μm corresponding to the dorso-medial cerebral cortex shown in A. The mean number of positive cells/total number of cells ± SEM was calculated from 4 sections of 5 different embryos of each genotype **C.** Quantification of the number of Sox2 and MAP2 positive cells per bin is shown in which the corresponding neocortex regions (VZ, SVZ, IZ and CP) are indicated. The mean number of positive cells ± SEM was calculated from 4 sections of 5 different embryos of each genotype. *p < 0.05; ***p < 0.001 in *c-fos*−/− embryos with respect to the *c-fos* +/+ condition as determined by two-way ANOVA with Bonferroni post-test.

Finally, we examined the expression of MAP2, a neuron-specific protein that stabilizes dendritic microtubules in post-mitotic neurons and allows the delimitation of the cortical plate within the developing cerebral cortex [32, 33]. The presence of Sox2 and MAP2 was established by immunofluorescence assays of E14.5 *c-fos* +/+ and *c-fos −/−* brain coronal slices (Figure 7A). As observed previously (Figure 6B), the thickness of the Sox2 positive zone did not differ significantly between both groups of embryos although more Sox2 positive cells were found both upon total cell counting and in the SVZ of the *c-fos −/−* with respect to the *c-fos +/+* mice (Figure 4A and Figure 7C). In the *c-fos −/−* embryo, the number of total MAP2 positive cells respect to the total number of cells (DAPI positive cells) was reduced (Figure 7B) and the distribution of these cells was clearly different in IZ and CP in comparison with that in the *c-fos +/+* embryos (Figure 7C).

**Figure 8:**
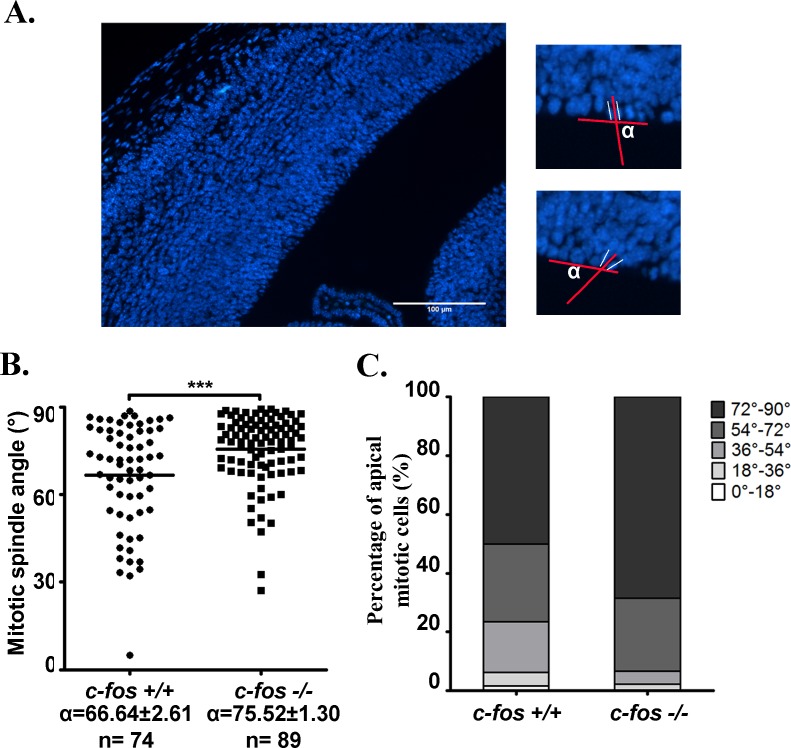
Distribution of the mitotic spindle angle in *c-fos* +/+ and *c-fos* −/− embryos **A.** Representative photomicrograph of a coronal cerebral cortex section stained with DAPI in which the angle of cells undergoing apical mitosis was measured (left panel). The right panel is the magnification of an example of cells undergoing: vertical (upper panel) and horizontal (lower panel) mitosis. **B.** The distribution of the cleavage plane of the mitotic angle was measured. Mean angle values (α) ± SEM were measured in 4 embryos of each genotype; n corresponds to the number of measurements performed. **C.** The angles measured for apical mitotic cells were distributed in 5 groups according to the cleavage plane angle in intervals of 18° each. Results are presented as percentage of total apical mitotic cells in each angle range for each genotype. ***p < 0.001 in *c-fos* −/− embryos with respect to the *c-fos* +/+ condition as determined by the unpaired t test with Welch's correction.

Next we measured the mitotic angle of cells undergoing apical mitosis, a parameter used to evaluate the fate of the daughter cells: cells undergoing differentiation predominantly show a horizontal mitotic angle whereas those determined for cell population expansion undergo mitosis with a vertical angle [34] (Figure 8A). It was found that a higher percentage of *c-fos −/−* progenitors present a predominant vertical mitotic angle that lies in the 72°-90° range in comparison with *c-fos* +/+ progenitors (68.54% versus 50.00% for *c-fos* −/− and *c-fos* +/+, respectively) (Figure 8B and 8C) indicating a preference of the *c-fos −/−* progenitor cell population to expand in detriment of their tendency to advance to neuronal differentiation.

**Figure 9:**
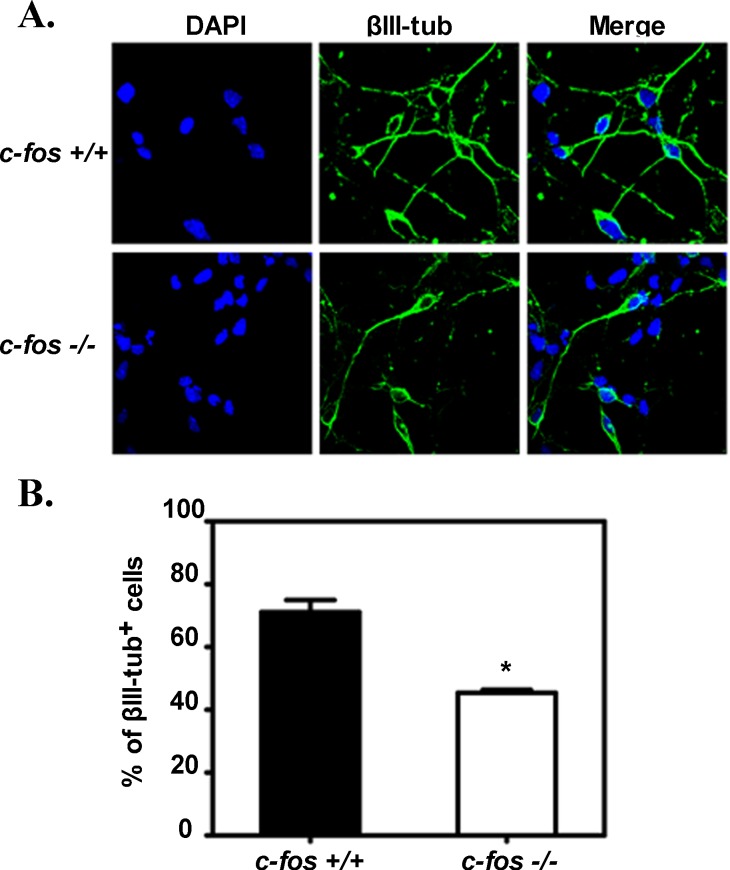
NSPCs from *c-fos* −/− cerebral cortex undergo less neuronal differentiation in the presence of NGF **A.** NSPCs isolated from cerebral cortex of E14.5 *c-fos* +/+ and *c-fos* −/− embryos induced to differentiate *in vitro* by culturing during five days in the presence of 40 ng/ml of NGF were analyzed by immunofluorescence for βIII-tubulin (green) and DAPI (blue) staining. **B.** Percentage of βIII-tubulin positive (+) cells shown in A. Results are the mean percentage values of cells showing positive staining for βIII-tubulin ± SEM calculated from the quantification of 10 fields from 3 different independent experiments. Number of cells with positive DAPI staining was considered as 100%. Statistical analysis: *p < 0.01 in NSPCs isolated from *c-fos −/−* embryos with respect to the *c-fos* +/+ condition as determined by unpaired t-test with Welch's correction.

Even if the reduction observed in the population of post-mitotic cells may be partially explained by an increase in the number of apoptotic cells in the *c-fos −/−* embryo, it was also considered if the capacity of the *c-fos −/−* progenitors to differentiate was affected. To examine this possibility, experiments were performed in cultured cells: NSPCs were isolated from cerebral cortex of E14.5 *c-fos* +/+ and *c-fos −/−* embryos, cultured for five days in the presence of Nerve Growth Factor (NGF) to induce neuronal differentiation and then immunostained for βIII-tubulin and DAPI (Figure 9A). A significantly smaller percentage of cells showed βIII-tubulin expression in the *c-fos −/−* condition than in the *c-fos* +/+ embryos (Figure 9B) with no differences in the rate of cell death between both genotypes (determined by Annexin V staining; data not shown).

**Figure 10:**
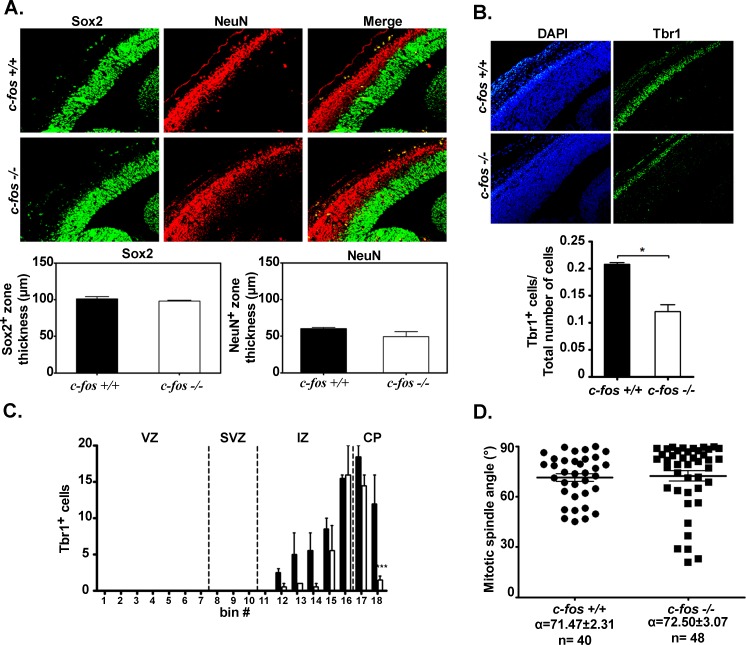
Cerebral cortex from E13.5 only shows differences in Tbr1 expression between *c-fos* −/− and *c-fos* +/+ embryos **A.** Representative photomicrographs of the dorso-medial cerebral wall from E13.5 *c-fos* +/+ and *c-fos* −/− embryos stained for Sox2 (green) and NeuN (red) (left panel). The corresponding quantification of the thickness of the Sox2 and NeuN positive zone are shown in the lower panel. **B.** Representative photomicrographs of the fluorescent staining for Tbr1 (right column, green) and for DAPI (left column, blue) in brain coronal sections from the dorsomedial cerebral wall of *c-fos* +/+ and *c-fos* −/− embryos. Quantification of the number of Tbr1 positive cells/total number of cells in a region of 100μmx180μm corresponding to the dorso-medial cerebral cortex is shown in the lower panel. Images were obtained with a fluorescence microscope (Olympus BX51) using a 40X objective. **C.** The histogram shows the quantification of the number of nuclei positive for Tbr1 staining/bin in brain coronal sections of E13.5 *c-fos* +/+ (black bars) and *c-fos −/−* (white bars) embryos, as indicated. Neocortex regions (VZ, SVZ, IZ and CP) are indicated. Results are the mean number of positive nuclei ± SEM of four quantifications from 4 consecutive sections of 4 embryos of each genotype. ***p <0.001 in *c-fos* −/− samples with respect to the *c-fos* +/+ condition as determined by two-way ANOVA with Bonferroni post-test. **D.** The distribution of the cleavage plane of the mitotic angle was measured. Mean angle values (α) ± SEM were calculated from 3 embryos of each genotype; n corresponds to the number of measurements performed.

To advance in determining at which developmental stage the differences between *c-fos* −/− and *c-fos* +/+ embryos can be observed at the cellular level, similar studies to those reported above with E14.5 were performed at E13.5. As shown in Figure 10, no significant differences were observed either in the total thickness of the cerebral cortex showing positive Sox2 or NeuN expression (Figure 10A) or in the mitotic spindle angle (Figure 10D). Significant differences were found in the total amount of Tbr1-positive cells respect to the total number of cells (DAPI positive cells) and in the distribution of these cells in the CP of E13.5 *c-fos −/−* embryos (Figure 10B and 10C), indicating that differences are starting to be apparent at this developmental stage.

In summary, taken together, the results show that: i) the differences in the final stage of development between *c-fos* +/+ and *c-fos −/−* embryo brains start to become apparent at early stages of embryonic development (E13.5) and remains evident up to the adult age of the animals; ii) this is a very complex phenomena that results from the concomitant occurrence of various events including an increased apoptotic rate and a reduced tendency of NSPCs to differentiate with the consequent reduced population of post-mitotic neurons.

### Phospholipid synthesis activation *vs* AP-1 content in the developing cerebral cortex

As stated, c-Fos has two distinct cellular functions that is, as an AP-1 transcription factor (nuclear activity), and as an activator of lipid synthesis (cytoplasmic activity) [15, 35]. Consequently, in an attempt to determine which of these activities of c-Fos are participating in the establishment of the differences seen within the developing cerebral cortex, we evaluated phospholipid labeling and AP-1 content in homogenates prepared from cerebral cortex of E14.5 *c-fos* +/+ and *c-fos −/−* embryos.

**Figure 11:**
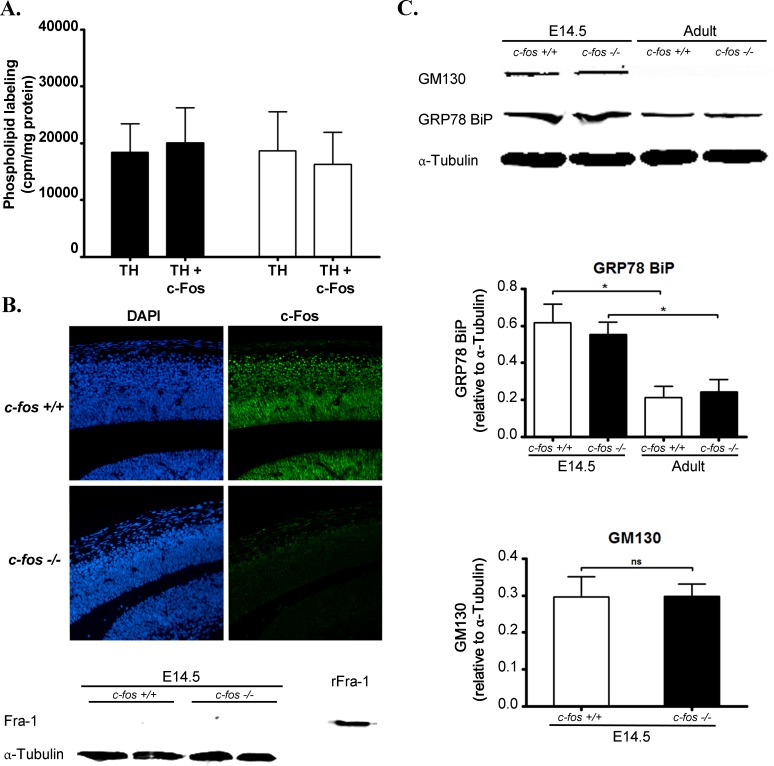
Phospholipid synthesis in the developing cerebral cortex **A.** Cerebral cortex of E14.5 *c-fos* +/+ (black bars) and *c-fos* −/− (white bars) embryos were used to prepare total homogenate (TH). TH´s were assayed for phospholipid synthesis capacity in the absence (TH) or presence (TH +c-Fos) of recombinant c-Fos at a final concentration of 1ng of c-Fos/μg of TH protein. Results are the mean cpm incorporated into phospholipids/mg protein ± SEM of 4 independent experiments performed in quadruplicate. **B.** Representative photomicrographs of the fluorescent staining for DAPI (left column, blue) and for c-Fos (right column, green) in brain coronal sections from the dorso-medial cerebral wall of *c-fos* +/+ and *c-fos* −/− embryos. Images were obtained with a fluorescence microscope (Olympus BX51) using a 40X objective. The bottom panel shows a Western blot for Fra-1 expression in total homogenate prepared from cerebral cortex of E14.5 *c-fos* +/+ (first 2 lanes) and *c-fos* −/− (lanes 3 and 4) embryos. The last lane corresponds to a sample of recombinant Fra-1 (rFra-1) used as a positive staining control. **C.** The expression of the Golgi marker GM130 (top row), of the ER marker GRP78 BiP and of α–Tubulin used as a loading control were determined by Western blot in cerebral cortex from E14.5 *c-fos* +/+ (first 2 lanes) and *c-fos* −/− (lanes 3 and 4) embryos and in *c-fos* +/+ and *c-fos* −/− adult cerebral cortex total homogenate. The levels of expression relative to α–Tubulin are shown in the histograms below for GRP78 BiP (top panel) and GM130 (bottom panel). The histogram represents the mean value ± SEM from five independent experiments. Note that levels of GM130 were below the level of detection in both *c-fos* +/+ and *c-fos* −/− adult samples whereas both *c-fos* +/+ and *c-fos* −/− embryos evidenced significantly higher relative GRP78 BiP expression with respect to adult animals. *p < 0.01 in embryos with respect to the adult as determined by two-way ANOVA with Bonferroni post-test.

*In vitro* phospholipid synthesis was determined using total homogenate from cerebral cortex of E14.5 *c-fos* +/+ and *c-fos −/−* embryos. No significant differences were observed in the labeling of total phospholipids between both groups of embryos in spite of the differences in the expression of c-Fos between the two groups of embryos: the *c-fos +/+* ones show high levels of c-Fos expression which clearly contrast with the lack of expression in the *c-fos −/−* embryos (Figure 11B). Moreover, addition of recombinant c-Fos to the assays did not enhance the incorporation of ^32^P from [γ^32^P]-ATP into phospholipids (Figure 11A). It has been found that only one other protein that is member of the Fos family proteins, Fra-1, has a similar capacity as c-Fos to activate phospholipid synthesis [19]. Consequently, Fra-1 expression was determined in cerebral cortex from E14.5 *c-fos* +/+ and *c-fos −/−* to establish if this protein was compensating the lack of c-Fos in the *ko* embryos. No expression of Fra-1 was found in either group (Figure 11B, lower panel), precluding an effect of this protein on lipid synthesis.

Previous studies have shown that at early embryonic stages of brain development in which high rates of proliferation occur, the content of Golgi and ER membranes is significantly higher than that in adult brain cells [36]. So, we examined the expression of GM130 as a marker of the Golgi apparatus and of GRP78 BiP as a marker of the ER in E14.5 *c-fos* +/+ and *c-fos −/−* embryos and adult mice brain. As expected, the relative content of GRP78 BiP significantly decreased in both *c-fos* +/+ and *c-fos −/−* adult brain samples as compared to either embryonic sample whereas the expression levels of GM130 were below the limit of detection in the adult animals (Figure 11C). In the light of these results, we hypothesize that membrane biogenesis rates are fully activated in both groups at the embryonic age assayed, thus masking any activating effect of exogenous c-Fos.

**Figure 12:**
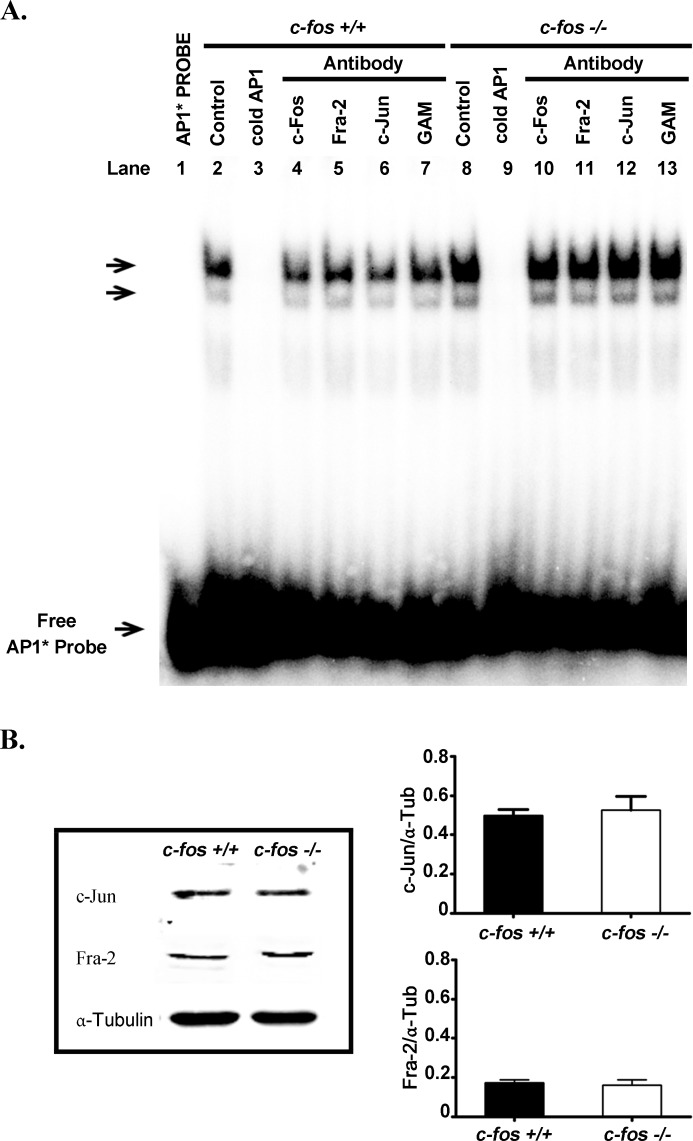
AP-1 content in the developing cerebral cortex **A.** EMSA determination of total AP-1 content in nuclear extracts prepared from cerebral cortex of E14.5 *c-fos* +/+ and *c-fos* −/− embryos. The arrow indicates the position of the AP1-DNA complex. Lanes 2 to 7 correspond to the *c-fos* +/+ and lanes 8 to 13 correspond to *c-fos* −/− embryos. Competition with cold AP-1 (100X) was assayed in lines 3 and 9. EMSA analyses to determine the composition of AP-1 complex were performed with antibodies against c-Fos (lane 4 and 10), Fra-2 (lane 5 and 11), c-Jun (lane 6 and 12) and a non-related antibody (goat anti mouse; GAM) (lane 7 and 13). Equal amounts of protein were loaded in each lane except lane 1 that contained no protein. Six independent assays were performed. **B.** Western Blot showing the expression of c-Jun, Fra-2 and α–Tubulin in total homogenate obtained from cerebral cortex of E14.5 *c-fos* +/+ and *c-fos* −/− embryos (left panel). Quantification of c-Jun (upper) and Fra-2 (lower) expression relative to α–Tubulin is shown (right panel). The results are the mean relative expression ± SEM of 5 samples for each genotype. No significant differences were observed between the *c-fos* +/+ and *c-fos* −/− conditions.

As c-Fos forms hetero-AP-1 transcription factor dimers mainly with c-Jun [12], EMSA assays were performed to examine these AP-1 dimers, using nuclear extracts from cerebral cortex of E14.5 *c-fos* +/+ and *c-fos −/−* embryos. It was found that the *c-fos −/−* samples have a tendency to contain more AP-1 dimers than that of *c-fos* +/+ animals (Figure 12A). These results suggest that the absence of *c-fos* results in a modification in the content of AP-1 dimers that could be partially responsible for the increased apoptosis and reduced neuronal differentiation observed in the *c-fos −/−* as compared with the *c-fos* +/+ developing cerebral cortex.

To determine if the composition of these AP-1 dimers differ between both groups of animals, c-Jun, Fra-2 and c-Fos antibodies were used for the EMSA assays. As can be observed in Figure 12A, the AP-1 dimers of the *c-fos* +/+ embryo contain c-Fos and c-Jun (in all cases, AP-1 labeling decreased in the presence of the c-Jun or the c-Fos antibody), while no labeling differences were observed in the presence or absence of Fra-2 antibody. By contrast, in the *c-fos −/−* embryo, no differences were found in AP-1 labeling upon addition of c-Jun or Fra-2 antibodies in spite that the *c-fos −/−* and the *c-fos* +/+ embryo contained similar amounts of c-Jun and Fra-2 protein as shown by the WB assay of Figure 12B. These results indicate that the lack of *c-fos* in the developing cerebral cortex of *c-fos −/−* embryos provoke a modification in the content and/or stability of the AP1 transcription factor dimers as compared to those of the *wt* condition.

## DISCUSSION

c-Fos, a well known AP-1 transcription factor, has been postulated to regulate key cellular events such as differentiation and growth [37]. However, the fact that *c-fos −/−* mice are viable evidences that this protein can be substituted by other members of this family of transcription factors, although its absence causes severe alterations in the development of the animal (infertility, osteopetrosis, hematologic and nervous system alterations) [10]. Caubet *J.F.* (1989) previously showed that c-Fos is expressed at different stages of nervous system development suggesting that the AP-1 family of transcription factors plays a role in the regulation of the coordinated expression of genes involved in the process [38]. However, considering that, in the past years it has been reported that in addition to its AP-1 activity, c-Fos has the capacity to activate lipid synthesis (reviewed in [35, 39]), it was deemed of interest to examine in more detail the role of c-Fos in the development of the CNS, and more specifically in the cortical neuroepithelium and also by means of which of its assigned activities it is participating. As shown, body weight and brain size are significantly smaller in mice that lack *c-fos* expression. In the E14.5 embryos, mitosis index did not evidence differences between embryonic developing brains of both genotypes although more cells undergoing apoptosis in VZ of *c-fos −/−* mice were observed. Furthermore, immunostaining of Sox2, a radial glial cells and early IPCs marker, is higher in the neocortex and particularly in defined regions of the SVZ (bin 9) of *c-fos −/−* embryos whereas Tbr2 immunostaining shows no differences between *c-fos −/−* and *c-fos +/+* embryos. Other markers of post-mitotic neurons (Tbr1, NeuN, MAP2 and βIII-tubulin) evidence a lower number of differentiating cells in *c-fos −/−* embryos compared to the *c-fos +/+* condition. In addition, a predominant vertical mitotic angle of *c-fos −/−* apical progenitors indicates a reduced trend to differentiate [34]. The finding that *c-fos −/−* mice have more apoptotic cells than the *c-fos +/+* ones on the ventricular surface is surprising if it is considered that at least in fully differentiated neurons, c-Fos results pro-apoptotic rather than anti-apoptotic [3, 40]. However, the role of c-Fos in apoptosis depends on its partner in the AP-1 complex [41]. It still remains to be determined if different combinations of AP-1 components or if different co-activators acting on the same AP-1-dependent promotor regulate specific genes differently. Clearly, in the case c-Fos participates in regulating apoptosis in differentiating and differentiated neurons, either situation can be occurring thus making it more difficult to draw clear conclusions with respect to the complex and varied physiological roles that c-Fos can exert.

In addition to the capacity of NSPCs to differentiate into mature neurons, previous works have also implicated apoptosis of progenitors of the cerebral cortex as a critical determinant of the final size of the neocortex [42]. The *c-fos −/−* mice present an enhanced rate of apoptosis at the VZ together with the content of more non-differentiated progenitor cells. These observations suggest that NSPCs present in the neuroepithelium of *c-fos −/−* mice are less capable of reaching a final stage of differentiation due to an enhancement of cellular apoptosis and a diminution in the rate of differentiation of *c-fos −/−* progenitors cells. In line with these observations, cultured NSPCs isolated from cerebral cortex of *c-fos −/−* embryos are less capable to differentiate than those of sister cultures isolated from littermate *c-fos +/+* embryos. It should be noted, however, that in addition to changes in apoptosis, the duration of the cell cycle is of key importance for the generation of the different neurons (numbers and types) of the cortical plate, so changes in this parameter can also promote alterations in the neurogenesis [43, 44].

To discern if the differences observed in the neocortex were due to c-Fos´s activity as a lipid synthesis activator or as an AP-1 transcription factor [35] both activities were measured at the embryonic developmental stage E14.5. No difference in the labeling of phospholipids was observed between both genotypes, even after the addition of recombinant c-Fos to the assay medium indicating that the membrane biosynthesis machinery produces membrane at a rate high enough to be independent of its activation by c-Fos. In line with this possibility is the observation that the content of endoplasmic reticulum and Golgi membranes, where the bulk of membrane lipid synthesis occurs, is significantly higher in developing brain that in adult brain (Figure 11C) [36].

Taking into consideration the results described previously, we evaluated the content of AP-1 transcription factors in *c-fos −/−* and *c-fos +/+* embryos. More AP-1/DNA complexes tend to be present in the *c-fos −/−* embryos than in the *c-fos* +/+ ones. The prototypical form of AP-1 is a heterodimer between Jun and c-Fos, a combination that is more stable and binds DNA more tightly than the Jun homodimer [45, 46]. As the lack of c-Fos does not affect c-Jun protein expression in the embryo (results shown herein and [47]), Jun homodimers potentially can be formed in *c-fos* mutants. However, no evidence was obtained indicating that Jun homodimers are replacing the Jun/Fos heterodimers in the *c-fos −/−* embryos. It must be concluded that homodimers evidently cannot replace the loss of c-Fos as evidenced by the phenotype of the *c-fos −/−* animals, highlighting the need of both proteins for mouse normal development and growth [10, 11]. An explanation for this simultaneous requirement of both transcription factors, in addition to a decreased stability of Jun homodimers with respect to Jun/Fos heterodimers, is the observation that the transactivation domains of Fos and Jun act synergistically to regulate genes more efficiently than the Jun homodimer [48], thus supporting that distinct and complementary biochemical functions of Fos and Jun are exerted as transcription regulators [49].

Jun AP-1 heterodimers can also be formed with other proteins of the Fos family and the resulting AP-1 dimers containing these other Fos family members usually have both overlapping as well as unique functions and in a tissue-specific way [8]. Herein, no expression of Fra-1 could be evidenced in either the *c-fos −/−* or the *c-fos* +/+ embryos; only Fra-2 expression was observed. However, no clear evidence was obtained that either *c-fos −/−* or *c-fos* +/+ embryos contained Fra-2-containing AP-1 dimers. It is still unknown whether different combinations of AP-1 components regulate specific neural genes differently or whether a single combination of AP-1 plays a more general role in various neural genes [50], thus rendering an even more complicated scenario for genetically tracking the multiplicity of AP-1 dimers and the outcome of their activity in mammalian cells. By no doubt, a more detailed study of the regulatory capacity of c-Fos-containing or c-Fos non-containing AP-1 transcriptional factors will help to understand the physiological differences observed in these mice.

## MATERIALS AND METHODS

### Animals

*c-fos −/−* animals were obtained by inbreeding of *c-fos* +/− mice from Jackson Laboratories [10]. The progeny obtained was genotyped by PCR using the following set of primers: FKO1 5´-GAGCAACTGAGAAGACTGGATAGAGCCGGC-3´) and FKO2 (5´-GGAGAGCCCATGCTGGAGAAGGAGTCG-3´) to amplify the *wt* allele of *c-fos* and FKO2 and PN1 primers (5´-GGCGAGGATCTCGTCGTGACC-3´) for *c-fos* mutant allele scoring.

### Ethics statement

All the animals were on a C57BL/6J background and grown under standard conditions. Investigation has been conducted in accordance with the ethical standards and according to the Declaration of Helsinki and with the standards stated in the Guide to the Care and Use of Experimental Animals published by the Canadian Council on Animal Care and have been approved by the local animal care committee. (Facultad de Ciencias Químicas, Universidad Nacional de Córdoba, Argentina, Exp. 15-99-39796).

### Nuclei quantification in the adult cerebral cortex

Seven adult mice of each genotype (*c-fos +/+* and *c-fos −/−* mice) were sacrificed by cervical dislocation, brains were excised, fixed in 4% buffered paraformaldehyde (PFA) and cerebral cortices were dissected at 15-30 days post-fixation. A suspension of nuclei was obtained by mechanical dissociation of the fixed cerebral cortices [51]. An aliquot of 30μl of the nuclear suspension was added to a 150μl mixture of PBS (phosphate buffered saline) and PI (propidium iodide) and PI positive nuclei were quantified by flow cytometry. All samples were processed in duplicate. Two-parameters, Side Scatter (SSC-A) and PI (PI-A) were used to establish a selection gate around the PI positive nuclei, thus avoiding the small debris in the samples [52].

### Immunofluorescence

Mice were sacrificed on day 14.5 of gestation (E14.5), brains were excised and fixed overnight at 4°C by immersion in 4% PFA. Then samples were transferred to PBS and tissues processed for paraffin embedding using a Tissue-Tek processor (VIP, Leica). For histological analysis, coronal sections (5μm) were obtained with a RM 2125 RT Leica microtome and mounted onto glass slides. Tissue sections were de-paraffinized, unmasked in citrate solution (pH6) and incubated in PBS supplemented with 7.5% goat serum plus 7.5% fetal bovine serum for 1h at room temperature. Slides were incubated overnight with the required primary antibodies at 4°C. The antibodies used were: anti-cleaved caspase-3 (1:200, rabbit, ref 9661; Cell Signaling), anti-Tbr2 (1:200, rabbit, ref ab23345, Abcam), anti-Tbr1 (1:200, rabbit, ref ab31940; Abcam), anti-Phospho-Histone H3 (Ser10) (1:200, mouse, ref 9706; Cell Signaling), anti-βIII-tubulin (TUJ1) (1:200, rabbit, ref MRB-435P, Covance), anti-MAP2 (1:200, mouse, ref MAB 3418, Chemicon), anti-Sox2 (1:200, mouse, ref 4900, Cell Signaling and 1:200, rabbit, ref AB5603, Chemicon).

To evaluate the differentiation capacity of NSPCs isolated form cerebral cortex, differentiated cells in culture were rinsed, fixed in 4% PFA and permeabilized with a 0.1% Triton X-100 solution during 10 minutes at room temperature. Saturation was achieved by incubation in PBS supplemented with 5% bovine serum albumin (BSA) for 1 h at room temperature. Cells were incubated overnight at 4°C with anti-βIII-tubulin (1:1000, rabbit, ref T2200, Sigma). After three washes with PBS, slides were incubated for 1 h with the appropriate secondary antibody conjugated to an Alexa fluorochrome (Alexa Fluor 594 or 488, 1:500, Life Technologies). Nuclear staining was achieved by incubation with 4′,6-diamidino-2-phenylindole (DAPI) to quantify apoptosis by the detection of pyknotic nuclei. Slides were mounted with Fluoromount (Southern Biotechnologies Associates) and tissue sections examined under a fluorescence microscope (Olympus BX51) with a 40X objective in three channels (pseudo-colored red, green and blue in the figures). For stained cultured cells, samples were examined using fluorescence microscope (Fluoview 300, Olympus) with a 60X objective. Images were analyzed with Photoshop software (Adobe).

### Measurement of cerebral cortex thickness

Cortical slices were analyzed in a standard sector of the dorso-medial cerebral wall as previously described [53, 54]. Briefly, total thickness of the cerebral cortex was determined using DAPI stained coronal brain sections (5μm, paraffin sections). Measurements were made in two different right-left regions of both hemispheres using sections at identical antero-posterior positions. Quadruplicate measurements of both hemispheres from each embryo were performed. Ten embryos of each genotype were analyzed.

### Quantification of mitotic and apoptotic cells

Mitotic figures in the dorso-medial cerebral walls were counted with respect to their relative location as: apical (surface mitosis) or basal (non-surface mitosis). Mitotic figure counts were obtained from the right and left hemispheres from 4-5 sections of each brain. The length of each sector analyzed was measured and results were expressed as number of nuclei Phospho Histone H3 (Phospho H3) positive/100μm of length [55]. Identification of apoptotic cells was achieved by staining with cleaved caspase-3 to detect pyknotic nuclei [56]. Positive cells were assigned according to their location to the: ventricular zone (VZ), subventricular zone (SVZ), intermediate zone (IZ) or cortical plate (CP) that were delineated using Sox2, Tbr2 and Tbr1 immuno-detection. It should be noted that the delimitation of the different zones of the developing cerebral cortex (VZ, SVZ, IZ and CP) were made taking in consideration *wt* mice. The length and thickness of each sector analyzed was measured and results expressed as number of cleaved caspase-3 positive cells/0,1mm^2^ along the developing cerebral cortex.

### Quantification of cellular markers

For the quantification of Sox2, Tbr2, Tbr1 and MAP2 positive cells, the sector analyzed was 100μm in its medial-lateral dimension and 5μm (corresponding to section thickness) in its rostro-caudal dimension; each sector was divided into bins of 10μm in height in its radial dimension. The bins were numbered and aligned such that the first bin was at the ventricular surface, with its long axis parallel to the ventricular border. The number of labeled cells was counted in each bin (Supplementary Figure 2). Three or four cortical slices were analyzed per animal. The number of animals used in each experiment is indicated in the corresponding figure legend. The thickness of the region corresponding to differentiated neuronal cells and progenitor cells committed to a neuronal fate was established using βIII-tubulin stained coronal brain sections (5μm, paraffin sections). Measurements were made in two different right-left regions of both hemispheres using sections located at identical antero-posterior positions. Quadruplicate measurements of both hemispheres from each embryo were performed.

### Spindle orientation of apical mitosis

The spindle orientation of cells undergoing apical mitosis was quantified as described previously [57, 58] using a specially developed macro. Orientation of sister chromatids was first defined by drawing a line connecting the top and the bottom of each chromatid. The cleavage plane was defined as the line bisecting both chromatids orientation lines. The plane of the apical surface was determined as a tangent line to the ventricular zone and the angle between the cleavage plane and the apical surface measure.

### *In vitro* neuronal differentiation

*In vitro* neuronal differentiation was performed as described previously [59]. For this, cortices from 14.5 day-old C57BL/6J mouse embryos were dissected under a binocular microscope, carefully freed of meninges and dissociated mechanically in DMEM (Gibco). The suspension was pelleted by centrifugation, washed three times with DMEM and cells cultured in DMEM-B27 (Gibco) containing 40ng/ml of recombinant nerve growth factor (NGF, Sigma). Twelve well plates coated with 10μg/ml of poly-D lysine were seeded at 1×10^6^ cells per well. Cultures were kept in a water-saturated incubator with a 95% air-5% CO_2_ atmosphere for 5 days and analyzed by immunofluorescence.

### Statistical analyses

These analyses were conducted with Graphpad Prism (Version 5.0c) using two-way ANOVA with Bonferroni post-test for quantification of cellular markers or unpaired t-test with Welch's correction for quantification of cerebral cortex thickness, mitosis and apoptosis with significances at *p < 0.05 or **p < 0.01 or ***p < 0.001.

### *In vitro* phospholipid labeling

*In vitro* phospholipid labeling was assayed as described previously [5]. Total homogenate from cerebral cortex of E14.5 *c-fos +/+* and *c-fos −/−* embryos (100μg) were incubated for 60 min at 37°C in medium containing at final volume of 80μL, 140mM NaCl, 4.5mM KCl, 0.5mM MgCl_2_, 5.6mM Glucose, 64mM HEPES Buffer pH7.4, 3μCi of [^32^P]ATP (specific activity 3000 Ci/mmol, PerkingElmer Life Sciences), in the presence or the absence of recombinant c-Fos (1ng/μg of total homogenate) protein re-suspended in 300mM Imidazol/8M Urea as indicated. Reactions were stopped by addition of TCA-PTA (5-0.5% respectively, final concentration) and phospholipid labeling quantified [60].

### Electrophoretic mobility shift assays (EMSA)

EMSA were performed as described [17]. Briefly, nuclear extracts from cerebral cortex of E14.5 *c-fos +/+* and *c-fos −/−* embryos were prepared as specified by Wang *et al.* [11] and stored at −70°C until use. Double stranded oligonucleotide containing the AP-1 consensus sequence TGAGTCA (5´-CGCTTGATGAGTCAGCCGGAA-3´) (Promega) was end-labeled with ^32^P-ATP using T4 polynucleotide kinase. AP-1 binding reaction, AP-1 competition assay and electrophoresis in non-denaturing polyacrylamide gels were performed as stated by the manufacturer (Promega). For detection of the components of the AP-1 complex we used 1 uL of anti c-Fos (sc-52, Santa Cruz), anti Fra-2 (sc-604, Santa Cruz), anti c-Jun (sc-1694, Santa Cruz) and 0.5 uL of goat anti mouse (A11031, Molecular Probes).

## SUPPLEMENTARY MATERIAL FIGURES


