# Supplementary material for: Brain development is impaired in c-fos −/− mice
Source: Oncotarget. 2015 Jun 19;6(19):16883–901. doi: 10.18632/oncotarget.4527 (PMC4621926; doi:10.18632/oncotarget.4527)
Supplement: Supplementary file 1 [file oncotarget-06-16883-s001.pdf]

Brain development is impaired in *c-fos* -/- mice

Supplementary Material

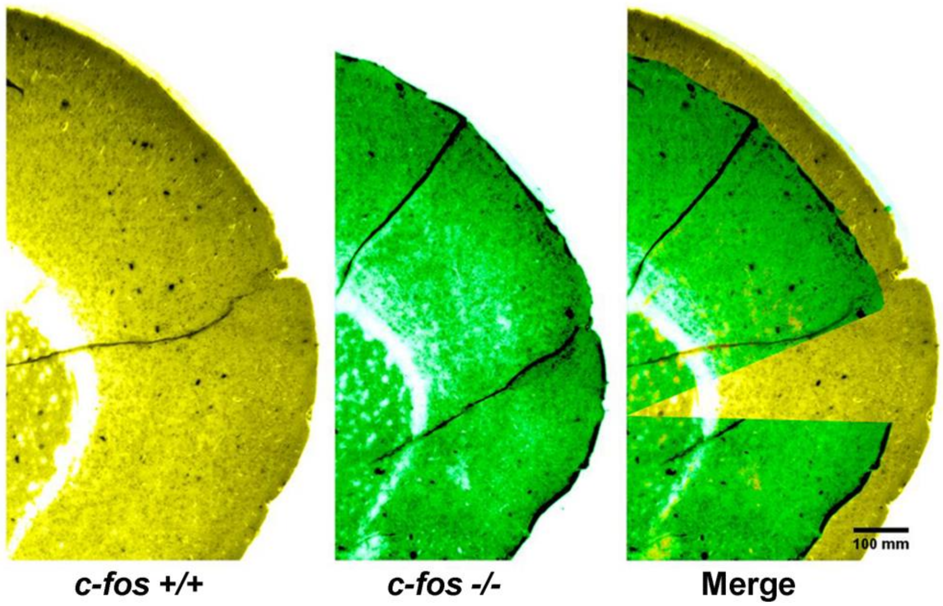

Supplementary Figure 1. Adult cerebral cortex thickness is reduced in *c-fos* -/- as compared to *c-fos* +/+ animals.

Representative photomicrographs of brain cortical slices from *c-fos* +/+ (left, pseudo colored green), *c-fos* -/- (middle, pseudo colored yellow) and the merged image (right) are shown. Images were obtained with a phase contrast microscope (Axioplan 135) using a 2,5 X objective. Scale bar: 100 mm. Note that in the merged image, part of the *c-fos* -/- image was cropped to better show the superposition of both images.

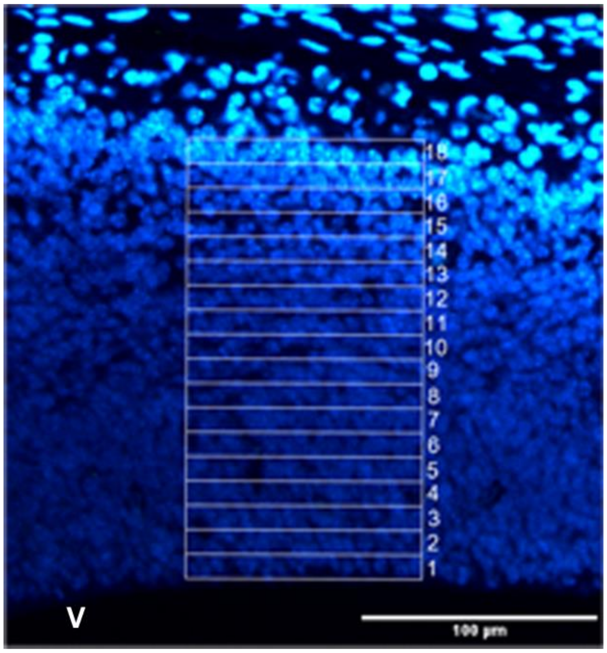

Supplementary Figure 2. Example of optical grid used for cortical slices examination. Cortical slices stained for DAPI (blue) were examined in a standard sector of the dorsomedial cerebral wall. The grid used has a sector of 100 µm in its mediallateral dimension and was divided into 18 bins of 10 µm of height in its radial dimension. The grid was aligned such that the first bin was at the ventricle (V) surface, with its long axis parallel to the ventricle border.

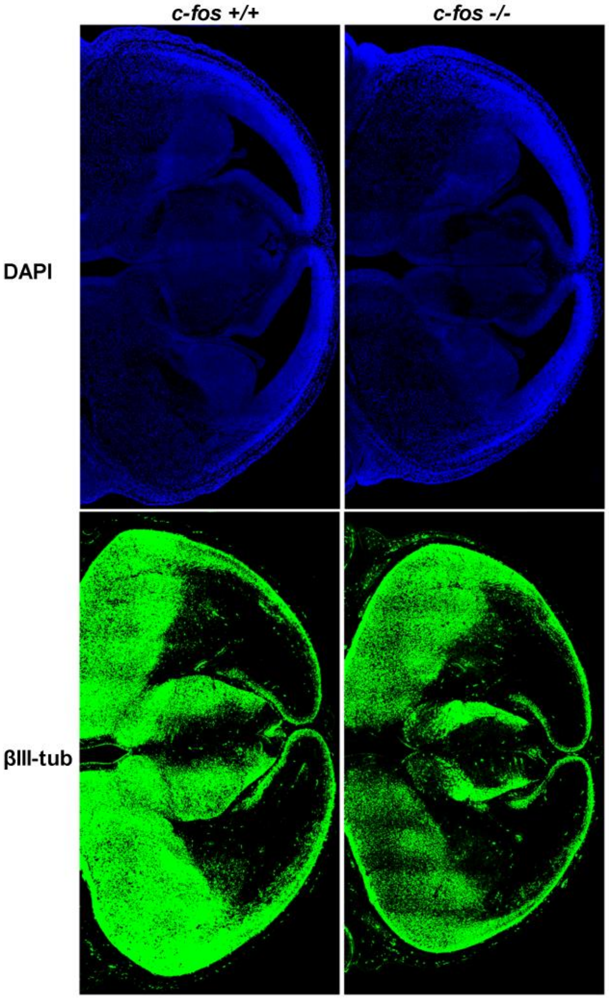

Supplementary Figure 3. *c-fos -/-* embryos shown a smaller βIII-tubulin positive region than the *c-fos* +/+ ones.

Representative photographs of the brain ventricle zone stained with DAPI (blue) (top row) and βIII-tubulin (green) (bottom row) corresponding to brain coronal sections of E14.5 *c-fos* +/+ and *c-fos* -/- embryos. Note the clear differences between *c-fos* +/+ and *c-fos* -/- embryos.

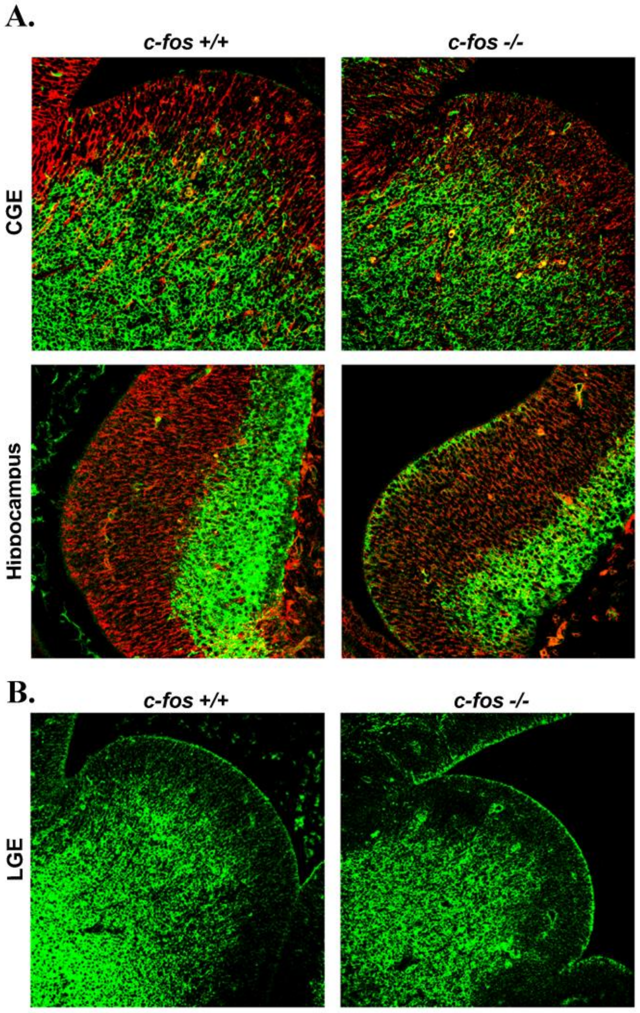

Supplementary Figure 4. E14.5 *c-fos-/-* cerebral slices show a clear reduction in the β-III tubulin positive zone at different areas of the brain.

A. Immunostaining for β-III tubulin (green) and Nestin (red) in brain coronal sections from E14.5 *c-fos* +/+ (left column) and *c-fos* -/- (right column) embryos. Caudal ganglionic eminence (CGE) and hippocampus were evaluated. Images were obtained with a fluorescence microscope (Olympus FV-1000) using a 40X objective. **B.** Immunostaining for β-III tubulin (green) in brain coronal sections from E14.5 *c-fos* +/+ (left column) and *c-fos* -/- (right column) embryos. Lower ganglionic eminence (LGE) was evaluated. Images were obtained with a fluorescence microscope (Olympus FV-1000) using a 20X objective.
